# Stretching Micro Metal Particles into Uniformly Dispersed and Sized Nanoparticles in Polymer

**DOI:** 10.1038/s41598-017-07788-3

**Published:** 2017-08-02

**Authors:** Abdolreza Javadi, Jingzhou Zhao, Chezheng Cao, Marta Pozuelo, Yingchao Yang, Injoo Hwang, Ting Chang Lin, Xiaochun Li

**Affiliations:** 1 Scifacturing Laboratory, Department of Mechanical and Aerospace Engineering, University of California, Los Angeles, CA 90095 USA; 2 Department of Materials Science and Engineering, University of California, Los Angeles, CA 90095 USA

## Abstract

There is a longstanding challenge to disperse metal nanoparticles uniformly in bulk polymers for widespread applications. Conventional scale-down techniques often are only able to shrink larger elements (such as microparticles and microfibers) into micro/nano-elements (i.e. nanoparticles and nanofibers) without much altering their relative spatial and size distributions. Here we show an unusual phenomenon that tin (Sn) microparticles with both poor size distribution and spatial dispersion were stretched into uniformly dispersed and sized Sn nanoparticles in polyethersulfone (PES) through a stack and draw technique in thermal drawing. It is believed that the capillary instability plays a crucial role during thermal drawing. This novel, inexpensive, and scalable method overcomes the longstanding challenge to produce bulk polymer-metal nanocomposites (PMNCs) with a uniform dispersion of metallic nano-elements.

## Introduction

Bulk PMNCs have drawn significant attention in the past two decades due to their unique physicochemical properties for functional applications^1–4^, including, but not limited to, electrically conducting polymers for transparent electrodes^5–7^, electromagnetic interface shielding and electrostatic dissipation^8^, plasmonic metamaterial as electromagnetic wave absorbers for solar cells^9–11^, and antimicrobial polymers^12^. *However, there is a long-standing challenge to disperse metal nanoparticles uniformly in bulk polymers*. Based on the nature of the incorporation of metal nanoparticles, the synthesis/fabrication methods can be divided into extrinsic and intrinsic^2^. For the extrinsic methods, nanoparticles are prepared separately and then incorporated into the polymer matrix during processing. Nanoparticles are to be dispersed via some kinetic approaches such as using shear forces or ultrasonic vibrations^8^. The surfaces of the metal nanoparticles are often functionalized or passivated to facilitate dispersion. A uniform dispersion of dense nanoparticles, however, is still hard to achieve. The extrinsic physical deposition methods are used to produce polymer-metal nanocomposites, but generally offer a homogeneous distribution only in thin films. The intrinsic methods are basically of chemical nature as metal particles are formed *in-situ* during processing. These wet chemical methods, generally very complex, can only produce a very limited set of polymer-metal nanocomposites with a somewhat reasonable dispersion of metal nanoparticles.

Scale-down techniques, such as extrusion and drawing, are often applied to proportionally shrink larger features into micro/nano-elements without much altering their relative spatial and size distributions. Thermal fiber drawing process has recently emerged as a high-temperature method for nanomanufacturing of some semiconductor and polymer^13–15^ nanoparticles out of long nanowires inside an amorphous cladding material, such as fused silica or thermoplastic polymers. But fabricating long metal nanowires has also been a long-standing problem due to capillary instability in thermal fiber drawing^16^.

This study is to explore thermal drawing of micro metal particles into nanoparticles in polymer matrix. We would expect that this scale-down technique can shrink the microparticles into nanoparticles without much altering their relative spatial and size distributions. As model materials, PES (with a glass transition point 225 °C) and Sn microparticles (with a melting point 231 °C) were used in thermal drawing. PES-Sn material system is of an interest for polymer-metal nanocomposite production through thermal fiber drawing technique. Our previous study showed the possible fabrication of the PES-Sn nanocomposites through thermal fiber drawing of typical core-cladding preforms^17^. After several consecutives thermal fiber drawings, these embedded uniform Sn wires were deformed and changed to Sn nanoparticles with non-uniform sizes. The previous study did not prove small crystalline Sn nanoparticles are uniformly dispersed and distributed in PES matrix. In addition, the typical core-cladding (Sn-PES) preform fabrication process is time-consuming since it consists of several steps such as machining deep holes to insert the Sn wires into the PES cladding and preform consolidation for 24 hour in vacuum furnace.

Here we show a novel and simple approach to fabricate PES-Sn preform, which needs substantially less time (about two hour) than the previous method. We also show that the Sn microparticles with random size distribution and poor spatial dispersion in PES were stretched into Sn nanoparticles with a much more uniform size distribution and spatial dispersion.

## Results

**Figure 1:**
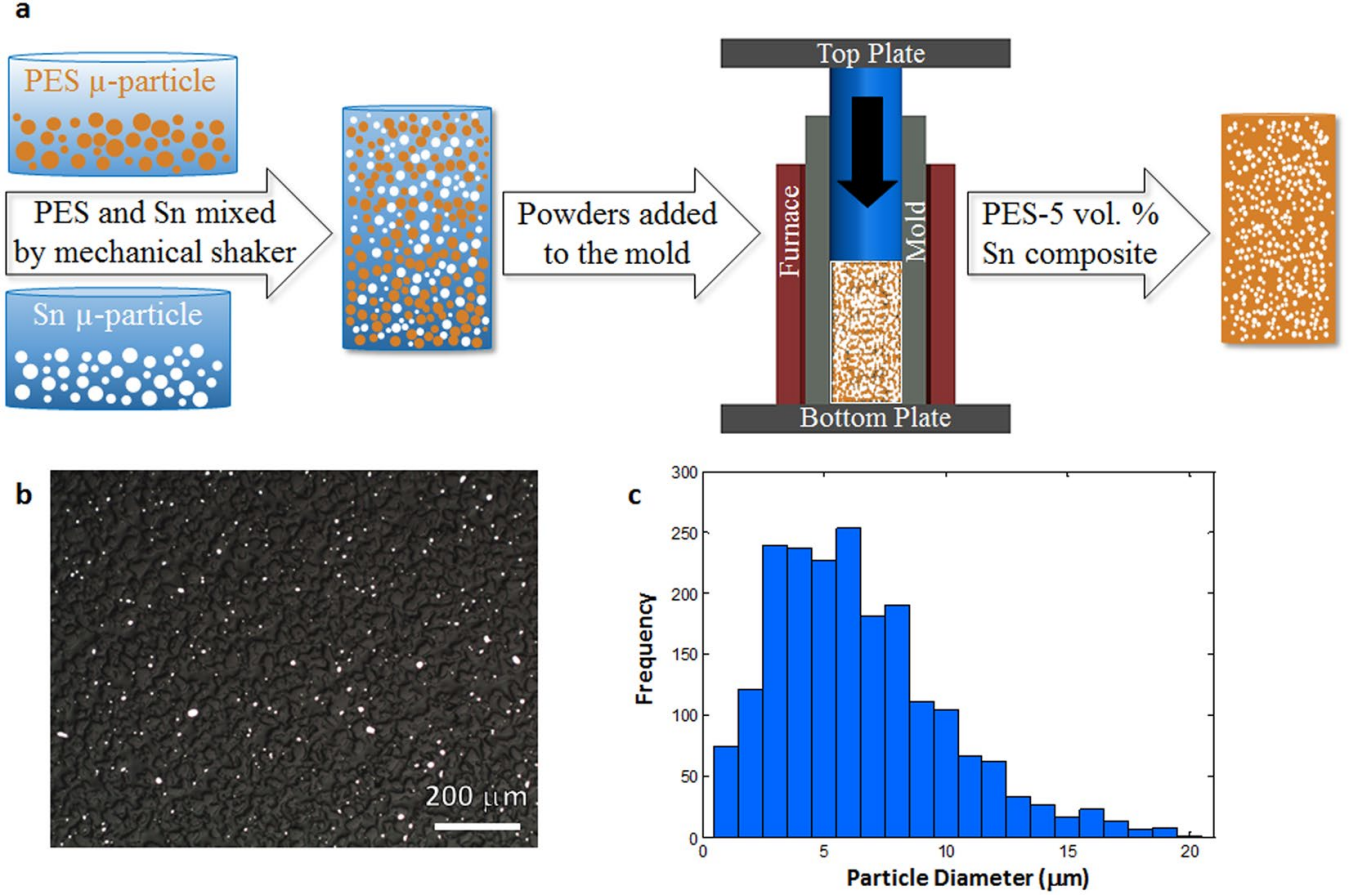
PES-5 Sn composite preform fabrication with poor metal distribution. (**a**) Schematic of the thermal consolidation process. (**b**) A typical optical microscope image from the longitudinal cross-section of a PES-5 Sn composite perform (scale bar: 40 μm). (**c**) Sn microparticles size distribution in a PES-5 Sn composite perform.

We first fabricated a composite preform of PES-5 volume percent tin (thereafter PES-5 Sn) via thermal consolidation (see Methods). Figure 1a represents schematic of the thermal consolidation process to fabricate the PES-5 Sn composite preforms. Figure 1b shows that the Sn microparticles are random in size and distribution in the PES preform while Fig. 1c presents the size distribution of the Sn microparticles in the PES-5 Sn composite preform.

**Figure 2:**
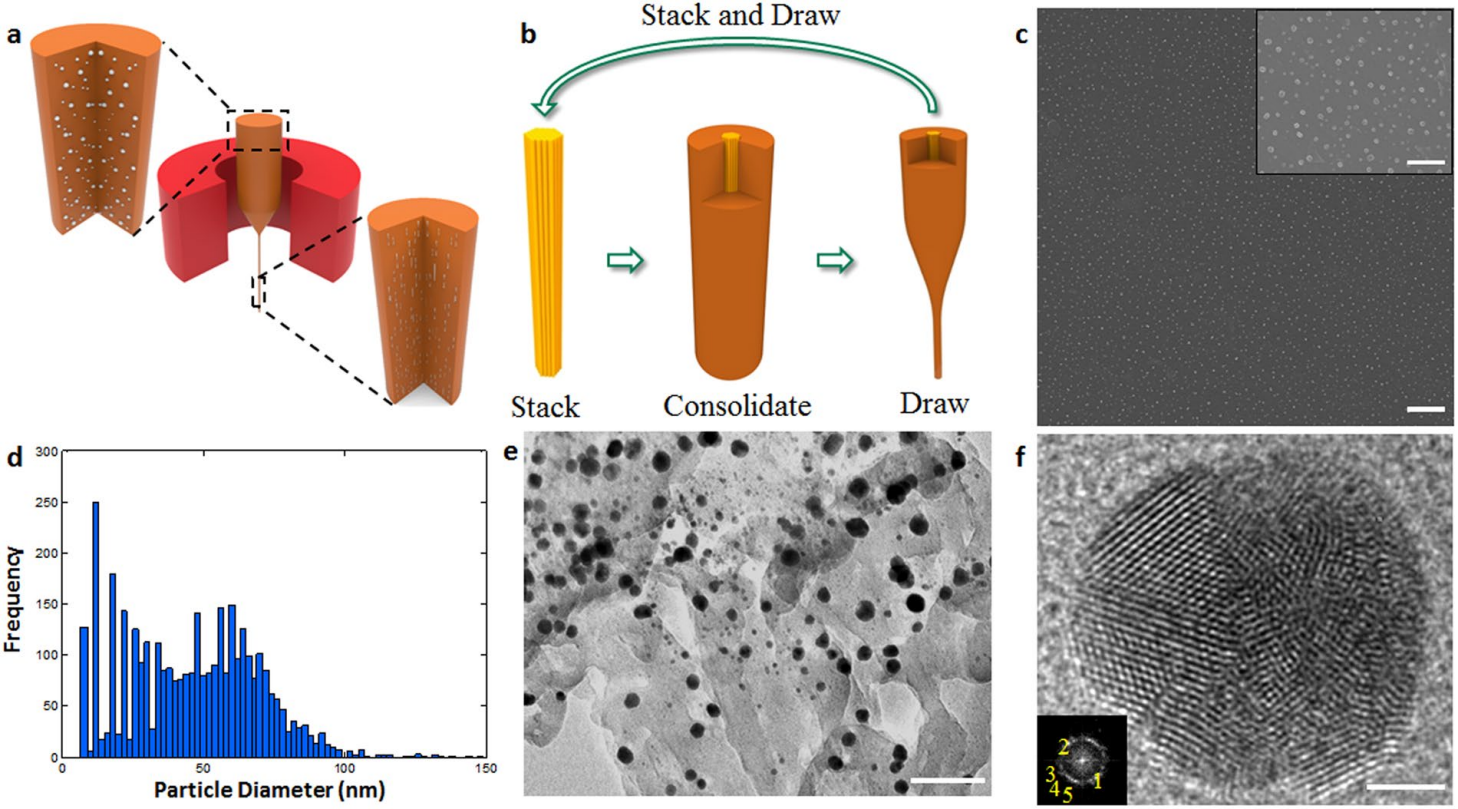
PES-5Sn nanocomposites by thermal drawing. (**a**) Schematic of the thermal drawing process. (**b**) Schematic of the stack and draw iteration. (**c**) SEM image of the longitudinal cross-section of PES-5Sn nanocomposite fiber with an inset to show high magnification (scale bar: 2 μm, inset: 500 nm). (**d**) Sn nanoparticle size distribution in PES-5Sn nanocomposites fiber (after the third cycle of the thermal drawing). (**e**) TEM image of Sn nanoparticles when PES cladding is dissolved after the third cycle of thermal drawing (scale bar: 50 nm). (**f**) Atomic resolution TEM image of Sn twinned-nanoparticles with polygonal shapes showing many facets as confirmed by their ring patterns (insets) (see Supplemental Table 2 for indexed diffraction patterns) (scale bar: 2 nm).

Then PES-5Sn composite preforms were thermally drawn at 300 °C to fabricate composite fibers (Fig. 2a). Drawn fibers (from the first cycle) was stacked and drawn (Fig. 2b) again for an additional cycle to obtain the PES-5Sn composite fibers. PES cladding was dissolved to reveal the Sn microfiber (See Materials and Methods). Using ImageJ software, the diameter of these Sn fibers was determined to be 6 ± 1 and 2 ± 0.5 μm after the first and second drawing cycles, respectively. It should be noted that the Sn microfibers vary in length while the diameter of the Sn fibers becoming smaller from first cycle to second cycle.

Drawn composite fibers (from the second cycle) was stacked and drawn (similarly to second drawing cycle) to fabricate PES-5Sn nanocomposite fibers with a uniform dispersion of Sn nanoparticles. Figure 2c shows SEM image of the PES-5Sn nanocomposite fiber after the third cycle of thermal drawing with uniform dispersion of Sn nanoparticles. More than 3500 nanoparticle measurements (over seven samples) were conducted to statistically determine the average size of the Sn nanoparticles to be 46 nm (Fig. 2d), noting that the smallest metal droplets reported via emulsification method were 600 nm, achieved by flow focusing of Hg in silicone oil, with a viscosity ratio of 0.004^18^. PES cladding was dissolved from the PMNC fibers to study the smallest Sn nanoparticle size (Fig. 2e) via transmission electron microscopy (TEM). Our results show that the smallest crystalline Sn nanoparticles produced were about 10 nm (Fig. 2f) which is in good agreement with theoretically predicted lower limit^19^.

## Discussion

**Figure 3:**
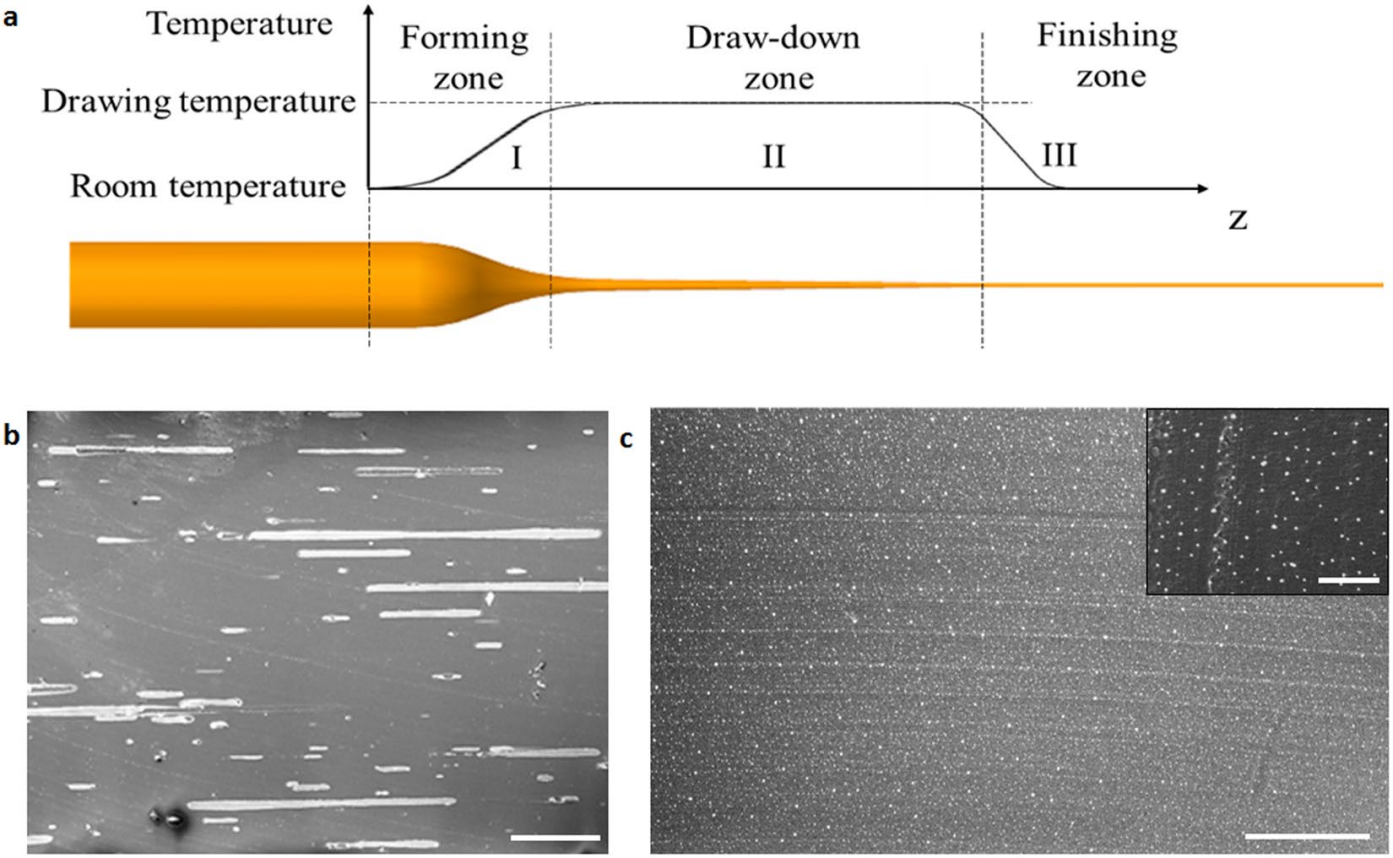
Metal droplet deformation during thermal drawing. (**a**) Evolution of metal droplet/wire during cyclic thermal drawing. (**b**) SEM image of the composite fiber longitudinal cross-section after several consecutive cuts were made on the fiber surface (after first thermal drawing cycle) (scale bar: 50 μm). (**c**) SEM images of a thin film cut from the PES-5Sn nanocomposite fiber (after the third cycle of the thermal drawing) with an inset to show high magnification (scale bar: 5 μm, inset: 1 μm).

During a steady state drawing, while the materials are fed into and pulled from the furnace at constant speeds, the shape and temperature profiles of the preform along its axis is steady. It is therefore customary to divide the regions where the fiber contraction occurs into three zones^20^, namely forming zone (I), draw-down zone (II), and finishing zone (III) as shown in Fig. 3a. It is believed that in the forming zone, a positive thermal gradient is present so that the preform becomes viscous as the temperature reaches its glass transition point and therefore starts to flow. The embedded metal wires/particles melt in this region and tend to remain as or break-up into droplets since the viscous shear stress is not yet large enough to overcome the surface tension force^13^. As the stress in the cladding flow grows stronger in the draw-down zone, the droplets are stretched to form liquid threads and droplets. The deformation ceases once the solidification temperature is reached, which freezes the liquid threads or droplets into their final shapes. In the first drawing cycle, Sn microfibers instead of particles were obtained (Fig. 3b) possibly because the microfibers solidified before the capillary instability grew Significantly. Subsequent break-up and stretching of these microfibers led to the results shown earlier in Fig. 2c and e. After the third drawing cycle, Sn nanoparticles are uniformly dispersed in the PES-5Sn nanocomposite fiber (Fig. 3c).

**Figure 4:**
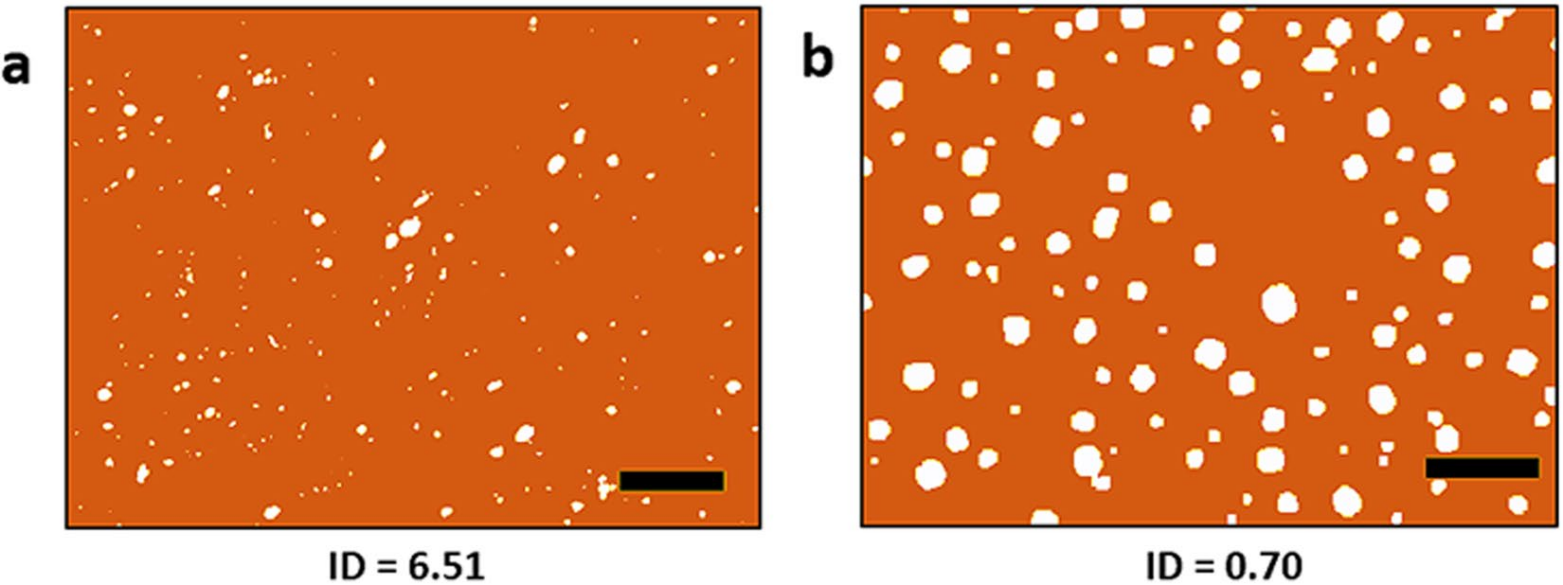
Binarized images and Index of Dispersion processed by ImageJ for spatial distribution analysis. (**a**) A typical optical microscope image from a longitudinal cross-section of the initial composite preform processed by ImageJ software (scale bar is 100 μm). (**b**) A typical SEM image of the longitudinal cross-section of the PES-5Sn nanocomposite fiber processed by ImageJ software (scale bar is 300 nm).

In addition to the observed micro-to-nano size reduction, statistical analysis of the optical microscope and SEM images (Fig. 4a and b) of the initial composite preform and PES-5Sn nanocomposite fiber longitudinal cross-sections show a transition of particle spatial distribution from non-uniform to uniform (details in Supplementary Information S1).

In summary, this work focused on the scalable nanomanufacturing of polymer-metal nanocomposites enabled by thermal drawing nano-emulsification. A simple, and cost-effective method was used to fabricate PES-5 Sn composite preforms. After the first thermal drawing cycle, metal microfibers with various diameters and lengths were well aligned in the longitudinal direction of composite polymer fibers. Using a stack and draw iteration, further drawing cycles produced polymer-metal (PES-5Sn) nanocomposite fibers with a uniform dispersion of Sn nanoparticles. Uniformly dispersed Sn nanoparticles were successfully obtained in the polymer fibers from non-uniform Sn microparticles for the first time. This scalable, novel, and inexpensive manufacturing method paves a new way for mass production of polymer-metal nanocomposites with well-dispersed nanoscale crystalline metal elements for widespread applications.

## Methods

### Composite preform fabrication

Non-uniform Sn (Sigma-Aldrich Corporation) and PES (Sumika Electronic Materials, Inc.) microparticles were used with an average diameter of 40 and 60 μm, respectively. The PES (95 vol.%) and Sn (5 vol.%) microparticles were first blended by a mechanical shaker for one hour. The well-blended microparticles were added to a cylindrical stainless steel mold with an outer diameter (OD) of 58 mm, an inner diameter (ID) of 19.05 mm, and a height of 152.4 mm. A hydraulic press was used to compact the well-blended powder mixtures at room temperature. An electrical resistance furnace was then used to sinter the compacted powders at 260 °C for one hour to form a solid preform of PES-5Sn composite preform.

### Thermal fiber drawing cycles

Thermal fiber drawing was used in a cyclic manner to gradually reduce the size of the Sn microparticles embedded in PES matrix down to nanometer size. In the first cycle of thermal drawing, the PES-5Sn composite preform with a diameter of 19.05 mm was drawn with 0.01 and 10 mm/s feeding speed and pulling speed respectively, down to a long composite fiber with an average diameter of 500 μm. The composite fibers from the first drawing cycle were cut (70 mm long), bundled and inserted into a cylindrical PES (with dimensions of 19.05 mm in OD, 3.8 mm in ID, and 80 mm in length) to form the preform for second drawing cycle. The preform for the third drawing cycle was fabricated following the same procedure. The second and third cycles of thermal drawings were carried out under the same conditions as in the first cycle.

### Characterization

To characterize the PES-5 Sn composite fiber (after first thermal drawing cycle), we cut five short fiber pieces (10 mm long each) from the long fibers. Fiber pieces were placed inside a rectangular box (25.4 × 25.4 × 5 mm made of stainless steel sheet cloth). The box containing the fiber pieces was gently submerged in an N-Methyl-2-pyrrolidone (NMP) solution bath for 5 hours at 70 °C to dissolve the PES claddings and expose the Sn microfibers (Supplemental Fig. 2). The aforementioned technique was repeated for the PES-5Sn composite fibers after the second cycle of thermal drawing.

After third drawing cycle, about 2 m long PES-5 Sn nanocomposite fiber was cut and placed in a glass beaker containing NMP. The beaker was heated to 70 °C for 5 hours to dissolve the PES cladding. The resultant solution was poured into multiple centrifuge glass tubes, which were then centrifuged for one hour leaving Sn nanoparticles at the bottom of the tubes. The unwanted liquid NMP was poured out from the tubes and fresh NMP was replenished in the glass tubes, and tubes were sonicated for 15 minutes. A micropipette was used to deposit few droplets of the solution on a TEM grid (Pure carbon 200 mesh, Ted Pella Inc.).

In addition, after each cycle of thermal drawing, composite fibers were studied from longitudinal direction. Fibers of 10 mm long was cut from the composite fiber and mounted on a carbon tape which was attached to a SEM stub from the other side (Supplemental Fig. 3a). Fibers were cut from its sidewall using an ultramicrotome tool (Supplemental Fig. 3b). In the case of third drawn composite fibers, films (100–500 nm thickness) were obtained by cutting from the fiber’s sidewall (in an area containing Sn nanoparticles) and manually placed on the carbon tape for SEM and high resolution TEM (HRTEM) study (Supplemental Fig. 4a and b).

## Electronic supplementary material

Supplementary Information
