# Supplementary material for: Stretching Micro Metal Particles into Uniformly Dispersed and Sized Nanoparticles in Polymer
Source: Sci Rep. 2017 Aug 2;7:7098. doi: 10.1038/s41598-017-07788-3 (PMC5540915; doi:10.1038/s41598-017-07788-3)
Supplement: Supplementary file 1 — Supplementary Information [file 41598_2017_7788_MOESM1_ESM.pdf]

Stretching Micro Metal Particles into Uniformly Dispersed and Sized Nanoparticles in Polymer

Abdolreza Javadi[1], Jingzhou Zhao[1], Chezheng Cao[2], Marta Pozuelo[2], Yingchao Yang[1], Injoo Hwang[1], Ting Chang Lin[1], and Xiaochun Li[1,2]

[1] Scifacturing Laboratory, Department of Mechanical and Aerospace Engineering, University of California, Los Angeles, CA 90095.

[2]Department of Materials Science and Engineering, University of California, Los Angeles, CA 90095.

Supplementary Information

S 1. Measurement of Index of dispersion

The index of dispersion (ID) is measured from [1],

$$ID = \frac{s^2}{\bar{x}} \quad (1)$$

Where $\bar{x}$ and s^2 are the sample mean and variance of the number of the points per quadrat. $ID < 1$ suggests uniform particle dispersion while $ID > 1$ indicates clustering. Image processing was done on each quadrat taken respectively at random locations from the longitudinal cross-sections of the PES-5Sn composite preform and PES-5Sn nanocomposite fiber. The quadrats counts are measured by ImageJ [2], shown in supplemental Fig 1.

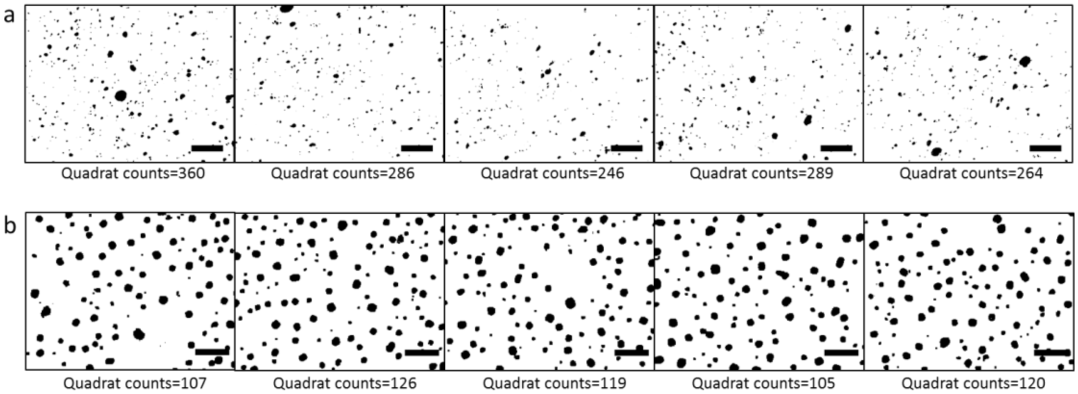

Supplemental Fig. 1 : a, optical microscope images taken from 5 random locations on the longitudinal cross-section of the PES-5 Sn composite preform processed by ImageJ software (scale bars are 100μm). **b**, SEM images taken from 5 random locations on the longitudinal cross-section of the PES-5Sn

nanocomposite fiber (after the third cycle of the thermal drawing) processed by ImageJ software (scale bars are 300nm).

The index of dispersion measured for the metal particle dispersion in the preform and the product fiber reduced from 6.51 to 0.70 (Supplemental Table 1), indicating the transition of metal particle dispersion from non-uniform to uniform.

Supplemental Table 1: Calculation of Index of Dispersion

Quadrat	1	2	3	4	5	Mean	Variance	ID
Preform	360	286	246	289	264	289	1881	6.51
Fiber	107	126	119	105	120	115.4	81.3	0.70

Supplemental Table 2: Indexed diffraction patterns

Spot number	d-spacing (nm)	β-Sn hkl	Intensity %	Error (%)
1	2.6	110	55	0.6
2	2.5	101	100	0.3
3	1.9	200	20	2.8
4	1.5	211	35	1.1
5	1.2	202	10	3.5

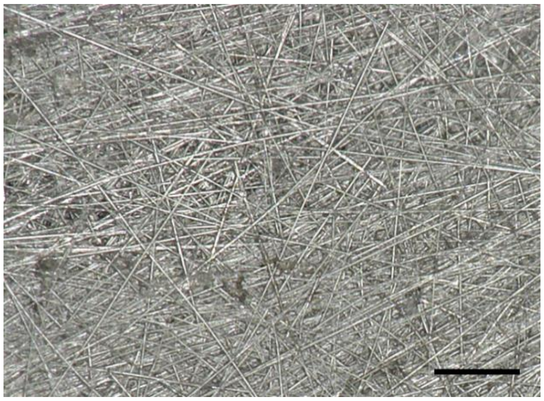

Supplemental Fig. 2: A typical optical microscope image taken after PES cladding was dissolved (after the first cycle of the thermal drawing) (scale bar: 200 μm).

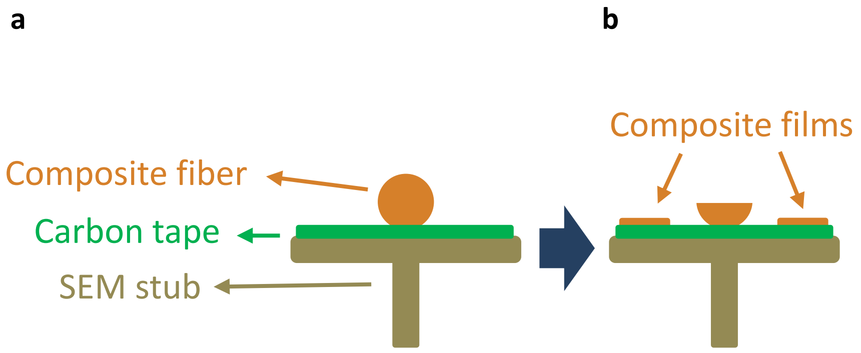

Supplemental Fig. 3: a, Schematic of a composite fiber attached to a carbon tape where the carbon tape is adhered to a SEM stub from the other side. **b**, Schematic of the study sample after several consecutive longitudinal cuts were made on PES-5Sn nanocomposite fiber and cut films were manually placed on the carbon tape for SEM and HRTEM study.

More composite films (with thickness ranging from 100 to 500 nm) were cut from the longitudinal side of the PES-5Sn nanocomposite fibers (after the third cycle of thermal drawing) for further investigation of Sn nanoparticles in PES. Composite films were carefully placed on a TEM grid (Pure carbon 200 mesh, Ted Pella Inc.). HRTEM confirms the existence of α-Sn and β-Sn and Sn_2O_3 nanoparticles embedded in PES matrix film.

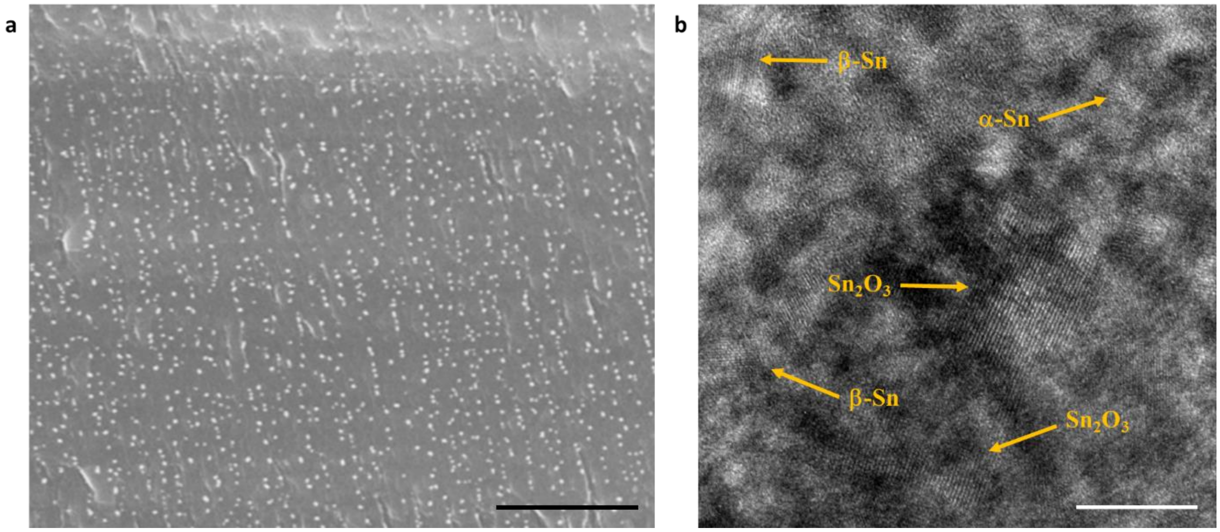

Supplemental Fig. 4: a, SEM image of a nanocomposite film cut from a PES-5Sn nanocomposite fiber prepared by ultramicrotome tool (after third cycle of thermal drawing) (scale bar: 2μm). **b**, HRTEM images of the nanocomposite film (scale bar: 10nm).

References

1. Zhou, Q., Zeng, L., DeCicco, M., Li, X. & Zhou, S. A comparative study on clustering indices for distribution uniformity of nanoparticles in metal matrix nanocomposites. *CIRP Journal of Manufacturing Science and Technology* **5**, 348-356 (2012).
2. Schneider, C. A., Rasband, W. S. & Eliceiri, K. W. NIH Image to ImageJ: 25 years of image analysis. *Nat Meth* **9**, 671-675 (2012).
